# Carrier screening in the reproductive setting—Are there medical implications for the heterozygote?—A guide for clinicians

**DOI:** 10.1002/pmf2.70280

**Published:** 2026-04-09

**Authors:** Emily B. Rosenfeld, Nicole Kasatkin, Bi Liu Yu, Milen Velinov, Justin S. Brandt, Elena Ashkinadze

**Affiliations:** ^1^ Division of Maternal‐Fetal Medicine Department of Obstetrics Gynecology, and Reproductive Sciences Rutgers Robert Wood Johnson Medical School New Brunswick New Jersey USA; ^2^ Division of Biostatistics and Epidemiology Department of Obstetrics Gynecology, and Reproductive Sciences Rutgers Robert Wood Johnson Medical School New Brunswick New Jersey USA; ^3^ Genetic Counseling Master's Program Rutgers University Piscataway New Jersey USA; ^4^ Department of Obstetrics and Gynecology Morristown Medical Center Morristown New Jersey USA; ^5^ Division of Genetics Department of Pediatrics Rutgers Robert Wood Johnson Medical School New Brunswick New Jersey USA; ^6^ Division of Maternal‐Fetal Medicine Department of Obstetrics and Gynecology NYU Grossman School of Medicine New York New York USA; ^7^ NYU Langone Health New York New York USA

**Keywords:** carrier screening panels, heterozygous, manifesting carrier

## Abstract

Carrier screening for genetic conditions performed preconception or during pregnancy allows identification of fetal risk for inherited autosomal recessive and X‐linked conditions. The goal is to identify at‐risk patients/couples and offer them reproductive options such as preimplantation genetic diagnosis, prenatal testing, or targeted newborn testing. Although individuals are expected to be carriers for autosomal recessive conditions, most couples are not carriers of the same condition. The traditional understanding is that carriers do not exhibit symptoms of disease. However, recent studies have shown that there may be clinical implications for medical management of some conditions in the carrier state, especially in the obstetrical setting. One study showed that 9% of patients who underwent large carrier screening panels were carriers for a condition with clinical implications in the heterozygous state. It is estimated that 2.5% of females are heterozygous for a condition that impacts their medical management during pregnancy. Obstetric clinicians who often interpret carrier screen results must be aware of the potential clinical implications and incorporate this information into their counseling and management plans. In this review, we examined three large panethnic, commercially available carrier screening panels that collectively cover 818 genes. We found that 112 (13.7%) of the conditions have definite or strong implications in the carrier state, and another 100 (12.2%) have moderate or limited evidence of implications in the carrier state. Of the identified genes, 19 may have an impact during pregnancy. This information, if used to guide clinicians on conditions that require further counseling and evaluation, may improve outcomes when incorporated into obstetric practice.

## 1 INTRODUCTION

Carrier screening has become an integral part of prenatal care [1, 2]. The purpose of carrier screening is to identify female patients who are carriers for X‐linked conditions and couples who are both carriers for an autosomal recessive condition [2, 3]. When a fetal risk is identified, this allows for reproductive options such as preimplantation genetic diagnosis or prenatal testing via procedures such as chorionic villus sampling or amniocentesis. The ability to screen hundreds of genes for variants has been made possible by next‐generation sequencing, which enables higher gene coverage and an increased detection rate [4, 5].

While the American College of Obstetricians and Gynecologists (ACOG) states that ethnicity‐based carrier screening and expanded carrier screening are acceptable strategies [2], the American College of Medical Genetics and Genomics (ACMG) recommends an ethnicity‐neutral approach to carrier screening to promote equity and inclusion [1]. The differences in testing recommendations between the two societies may partly reflect the demands of counseling for obstetricians. Nevertheless, carrier screening has become an integral part of prenatal care. Identification of potential fetal risk during the preconception period allows for reproductive interventions such as in vitro fertilization with preimplantation genetic testing for monogenic/single gene defects, which provides for a higher likelihood of having an unaffected child.

Traditionally, pretest counseling for carrier screening includes the purpose of the screen and setting the expectation that carrier status will likely be identified. However, for most conditions, patients can be reassured that carrier status alone rarely has medical implications, as for most autosomal recessive conditions, both alleles need to harbor a variant to have a phenotype. However, it has recently been recognized that carriers (heterozygotes) may be at risk for clinical manifestations that impact medical management [6]. For example, a carrier for ataxia telangiectasia (ATM gene) has a moderately increased risk for breast cancer and possibly other cancers [7]. Females who are carriers for a Duchenne's and Becker's Muscular Dystrophy (DMD) variant, which is an X‐linked condition, may manifest symptoms, particularly a dilated cardiomyopathy, that may go unrecognized but are exacerbated during pregnancy [8, 9, 10, 11].

In a retrospective cohort study of 4685 people who underwent a large carrier screening panel, 9% of subjects were at risk for clinical features in the carrier state alone [6]. Particularly relevant to an obstetrical practice, reproductive carrier screening identified 1 in 40 female patients whose carrier state was associated with clinical manifestations relevant to pregnancy [12].

Reproductive carrier screening is often ordered by obstetricians and reproductive endocrinologists who need to interpret the results for reproductive risk assessment. However, they also need to be aware that carrier status alone may have significant clinical implications that can impact medical management, even in the obstetrical setting. To our knowledge, there are currently no guidelines or an exhaustive list of genes known to have implications in the carrier state. The goal of this review is to assess the largest commercially available panels and identify conditions with clinical implications in the heterozygous state. This review provides a current overview and counseling strategies for patients who are found to be carriers of an autosomal recessive condition that causes clinical manifestations and/or an X‐linked condition in females, which can manifest with clinical features that may impact their medical management, particularly obstetrical management.

## 2 METHODS

We identified three large panethnic panels currently available via commercial laboratories. The three panels selected are: (1) Beacon 787‐Expanded by Fulgent Genetics includes 787 autosomal recessive and X‐linked disorders [13]. (2) Horizon Advanced Carrier Screening by Natera includes screening for 613 conditions [14]. (3) Inheritest 500 PLUS Panel by LabCorp includes 578 genes [15]. After removing duplicate genes across the three panels, 818 unique genes remained [13, 14, 15]. The first three authors conducted a comprehensive literature search to identify which of the 818 genes had at least one publication suggesting that carriers may exhibit clinical implications. The literature review was performed using PubMed with the gene name and keywords such as “heterozygote,” and “manifesting carrier.” Literature search was originally performed from April 15 to May 28, 2024 and all manuscripts were reviewed by an author. Two of the senior authors reviewed all findings identified by the first three authors. Levels of supporting evidence were determined by a medical geneticist based on the Clinical Genome Resource framework and classified each gene as “Definitive,” “Strong,” “Moderate,” “Limited,” “No Reported Evidence,” or “Conflicting Evidence” [16]. The medical implications were then organized by mode of inheritance, and obstetrical implications were further highlighted, as we anticipated that this publication would be targeted to clinicians working in the reproductive setting. A minimum of three cases is reported for each highlighted variant (except where specified). Publications with only one or two cases were excluded, as well as cases with compound heterozygotes. When available, the incidence of manifesting features is described in the text.

### 2.1 Conditions with known implications for carriers

Of the 818 conditions on the three most extensive expanded carrier screening panels at the time of publication, 212 of the 818 (25.9%) genes reviewed had clinical implications reported in the heterozygote state. Of the 90 X‐linked conditions, 75 (83.3%) have been reported to have potential implications for the female carrier.

A comprehensive list of 74 conditions is listed in Table 1. This table includes only conditions classified as definitive or strong by the Clinical Genome Resource framework. Definitive rating requires repeated evidence of the gene's role over time, defined as at least 3 years. Strong supportive evidence must include at least two separate studies showing the gene's role in disease, with unrelated probands and disease‐causality, and gene‐level evidence from different types of supporting experimental data. Both strong and definitive cases must have no contradicting evidence [16].

**TABLE 1:** Genes that have definitive or strong evidence of causing risk for the carrier in the heterozygote state. All genes are autosomal recessive, except where it is specified that there are autosomal dominant forms for the gene. Carrier frequency is described for the general population except where a specific ethnicity is described.

Condition (gene)	Pregnancy concerns/lifetime implications/clinical guidance	Carrier frequency (ethnicity) [13, 17]	Supportive evidence level [16]
Aicardi–Goutieres syndrome 1 (TREX1) [18, 19, 20, 21, 22]	*Lifetime implications*: Depending on the variant, individuals can have a risk of developing late‐onset lupus or retinal vasculopathy with cerebral leukodystrophy.	<1 in 500	Definite
Alpha‐1 antitrypsin deficiency (SERPINA1) [23, 24]	*Lifetime implications*: While most heterozygotes are asymptomatic if they are nonsmokers, carriers of PI*MZ pathogenic variant who are smokers are at increased risk for emphysema. *Clinical guidance*: Recommend tobacco cessation.	1 in 33 Caucasian/European: 1 in 19	Definite
Andermann syndrome (SLC12A6) [25, 26]	*Lifetime implications*: Heterozygotes may present with motor sensory neuropathy and agenesis of the corpus callosum.	<1 in 500 French Canadian: 1 in 23	Definite
Ataxia‐telangiectasia (ATM) [27, 28, 29, 30]	*Lifetime implications*: Carriers of the c.7271T > G variant have a breast cancer risk similar to BRCA mutation carriers. Absolute risk of developing breast cancer is 20%–30%, epithelial ovarian cancer is 2%–3%, and pancreatic cancer is 5%–10% for carriers. There is also an increased risk reported for neurodegenerative diseases, diabetes mellitus, and cardiovascular disease. *Clinical guidance*: Female heterozygotes with the c.7271T > G variant should begin breast cancer screening at age 25. Currently, there is no data on the benefit of risk‐reducing surgery. In addition, there is no current guidance on screening for other cancers.	1 in 100	Definite
Autoimmune polyendocrinopathy syndrome type I (AIRE) [31, 32, 33]	*Lifetime implications*: Variants can range from asymptomatic to immunodeficiency. *Clinical guidance*: APS‐1‐specific autoantibodies can inform if there are pathogenic AIRE variants.	1 in 150 Finnish: 1 in 79	Definite
Autosomal Recessive Congenital Myotonia (CLCN1)^a^ [34, 35]	*Lifetime implications*: Some of the CLCN1 variants are associated with autosomal dominant congenital myotonia and are associated with less severe muscle stiffness compared to homozygotes for the variants. Penetration and phenotype can vary within the family. *Clinical guidance*: Sodium channel blockers may relieve muscle stiffness in some patients.	1 in 176	Definite
Bernard–Soulier syndrome type A1 (GP1BA) [36, 37]	*Lifetime implications*: Carriers can have macrothrombocytopenia with mild symptoms. However, there is typically normal platelet function and normal megakaryocyte count.	<1 in 500	Definite
Butyrylcholinesterase deficiency (BCHE) [38]	*Lifetime implications*: This form of pseudochlolinesterase deficiency is associated with decreased clearance of succinylcholine in standard dosing for neuromuscular blockage. *Clinical guidance*: Anesthesiologists should be aware of any heterozygotes who are carriers for this condition as they may require adjustments in dosing of succinylcholine.	1 in 28	Strong
Carnitine palmitoyltransferase 1A deficiency (CPT1A) [39, 40, 41]	*Pregnancy concerns*: If the fetus is homozygous for this condition, then there is an increased maternal risk of acute fatty liver.	1 in 354 Hutterite: 1 in 16	Strong
Cerebral creatine deficiency syndrome 3 (GATM) [42]	*Lifetime implications*: In carriers, there have been noted renal proximal tubular mitochondrial issues that result in renal fibrosis that may lead to renal failure.	<1 in 500	Definite
Charcot–Marie–Tooth disease, GDAP1‐related (GDAP1) [43, 44]	*Lifetime implications*: Some of the genetic variants have mild forms of the syndrome in the carrier state and can present with neuropathy.	1 in 152	Definite
COL11A2‐related disorders (COL11A2) [45, 46, 47]	*Lifetime implications*: There are reports of heterozygotes having mild features of the condition including hearing loss, cataracts, myopia, slightly short stature, and joint pain.	<1 in 500	Definite
Combined pituitary hormone deficiency (POU1F1) [48, 49]	*Lifetime implications*: Carriers can present with either isolated or combined pituitary hormone deficiency.	<1 in 500	Definite
Congenital adrenal hyperplasia due to 11‐beta‐hydroxylase deficiency (CYP11B1) [50]	*Lifetime implications*: Children often present with hypertension. However, hypokalemia is rare. *Clinical guidance*: Adrenocorticotropin can be suppressed with glucocorticoids.	1 in 158 Moroccan Jewish: 1 in 35	Definite
Congenital hypothyroidism, TSHR‐related (TSHR) [51, 52]	*Lifetime implications*: Carriers can present with hyperthyroidism. *Clinical guidance*: Some heterozygotes do not respond to medical treatment.	1 in 500	Definite
Costeff syndrome (OPA3) [53, 54, 55, 56]	*Lifetime implications*: Heterozygotes of some variants have optic atrophy that can lead to loss of central or paracentral visual fields and color vision. It can sometimes be accompanied by peripheral neuropathy or gastrointestinal dysmotility.	<1 in 500 Iraqi Jewish: 1 in 50	Definite
Cystic fibrosis (CFTR) [57, 58, 59, 60, 61, 62, 63]	*Lifetime implications*: Heterozygotes may be at an increased risk for pulmonary and pancreatic complications.	1 in 32 African/African American: 1 in 61 Ashkenazi Jewish: 1 in 24 Caucasian/European: 1 in 25 East Asian: 1 in 94 Latino: 1 in 58	Definite
Cystinuria, non‐type I (SLC7A9) [64]	*Lifetime implications*: Carriers are at increased risk of cystinuria and nephrolithiasis, which may present in infancy or childhood. *Clinical guidance*: Incidence of nephrolithiasis may be decreased with modifications in diet and pharmacologic treatment.	1 in 42	Definite
Cystinuria, type 1 (SLC3A1)^a^ [65, 66, 67]	*Lifetime implications*: Some variants can present in an autosomal dominant fashion with incomplete penetrance. On prenatal ultrasound, fetuses may have hyperechoic colon. There is a lifetime risk of cystinuria and nephrolithiasis. *Clinical guidance*: Nephrolithiasis can often be managed through dietary modifications.	1 in 2451 Caucasian/European: 1 in 2051	Definite
Distal renal tubular acidosis (SLC4A1)^a^ [68, 69, 70, 71, 72, 73]	*Lifetime implications*: Heterozygous carriers can have kidney stones, mild renal acidification defects, and hypercalciuria. Certain variants are inherited as an autosomal dominant and heterozygotes present with incomplete distal renal tubular acidosis. There are also reports of spherocytosis, which may require a splenectomy in some patients.	<1 in 500	Definite
Du Pan syndrome (GDF5) [74, 75, 76, 77, 78, 79]	*Lifetime implications*: Most commonly, heterozygotes can present with brachydactyly. Rarely, some variants can have progressive symphalangism, deafness, tarsal/carpal fusion, or mild facial dysmorphism.	<1 in 500	Definite
Dyskeratosis congenita type 4 (TERT) [80, 81]	*Lifetime implications*: There is an increased risk of both congenital and age‐related pulmonary disease that may present earlier in life. There are some reports of increased risk for malignancy.	<1 in 500	Definite
Dyskeratosis congenita type 5 (RTEL1) [82, 83, 84]	*Lifetime implications*: Heterozygotes may be at risk of familial pulmonary fibrosis. A triad of dysplastic nails, abnormal skin pigmentation, and oral leukoplakia has been reported in some heterozygotes. These patients are at increased risk of cancer predisposition due to the shortened telomeres. *Clinical Guidance*: Heterozygotes should avoid environmental exposures such as smoking or inhalation of fibrogenic dust.	1 in 500 Ashkenazi Jewish: 1 in 203	Definite
Dystrophic epidermolysis bullosa (COL7A1) [85, 86]	*Lifetime implications*: Depending on the variant, different collagen types can be impaired, which can result in epidermolysis bullosa.	1 in 196	Definite
Factor V deficiency (F5) [87, 88]	*Lifetime implications*: Heterozygotes for Factor V Leiden without a personal or family history of venous thromboembolism have a 0.5%–3.1% risk of having a venous thromboembolism during pregnancy, and if they have a personal or family history of venous thromboembolism, the risk increases to 10%. Sequencing of this gene may also reveal Factor V deficiency, which can present as either a pro‐thrombotic or a bleeding disorder, depending on the variant. *Pregnancy considerations*: Heterozygotes for Factor V Leiden without a previous venous thromboembolism may warrant postpartum anticoagulation if there are additional risk factors. If a heterozygote has a first‐degree relative with a history of venous thromboembolism, antepartum anticoagulation can be considered, and postpartum anticoagulation is recommended. In carriers with a personal history of venous thromboembolism, they should receive antepartum and postpartum anticoagulation. Dosing recommendations are detailed in societal guidelines.	1 in 36 Caucasian/European: 1 in 19 Latino: 1 in 45 African/African American: 1 in 83 East Asian: 1 in 222 Native American: 1 in 80	Definite
Factor XI deficiency (F11) [89, 90]	*Lifetime implications*: Heterozygotes have an increased risk of bleeding, similar to that of homozygotes. *Clinical guidance*: Factor XI levels increase in pregnancy and drop postpartum. Heterozygotes should have factor XI activity assessed before delivery. They may need factor replacement prior to delivery or in the postpartum period. *Pregnancy concerns*: These heterozygotes are at increased risk of bleeding and should be co‐managed by hematology. Factor XI levels should be monitored during the third trimester, and replacement may be necessary during delivery or the postpartum period.	1 in 500 Ashkenazi Jewish: 1 in 11	Definite
Familial hypercholesterolemia (LDLR)^b^ [91, 92, 93, 94, 95]	*Lifetime implications*: Affected individuals typically have significantly elevated low‐density lipoprotein cholesterol, which can lead to early onset cardiovascular events. *Clinical guidance*: Pharmacotherapy is recommended, and patients should be encouraged to maintain a heart‐healthy diet. *Pregnancy concerns*: Pregnant females should be counseled on lifestyle changes, including smoking cessation, low‐saturated fat intake, and high dietary soluble fiber intake. Bile acid‐binding resins or LDL apheresis can be continued during pregnancy.	1 in 8 Amish: 1 in 2 Caucasian/European: 1 in 7 French Canadian: 1 in 8	Definite
Familial hyperinsulinism (ABCC8)^a^ [96, 97]	*Lifetime implications*: Heterozygotes may present with maturity‐onset diabetes of the young, which is responsive to sulfonylureas. *Clinical guidance*: If heterozygotes have diabetes, they typically can be treated with sulfonylureas.	1 in 112 Ashkenazi Jewish: 1 in 44 Finnish: 1 in 25 Middle‐Eastern: 1 in 25	Definite
Familial lipoprotein lipase deficiency (LPL) [98, 99, 100, 101]	*Lifetime implications*: Heterozygotes with additional risk factors are at an increased risk for premature atherosclerosis and have mildly elevated plasma triglyceride concentrations. *Clinical guidance*: Measurement of triglyceride levels and nutrition counseling.	1 in 500 French Canadian: 1 in 46	Definite
Familial Mediterranean fever (MEFV) [102, 103, 104]	*Lifetime implications*: Carriers may present with a broad spectrum of symptoms from classic to mild familial Mediterranean fever. Heterozygotes typically have a milder form of disease with a later age of onset (mean onset around 18 years of age). Depending on the variant, AA amyloidosis can occur in as high as 14%. *Clinical guidance*: Symptomatic carriers should be treated with colchicine and typically have an excellent response.	1 in 20 Mediterranean: 1 in 7	Definite
Fanconi Anemia, Group J (BRIP1) [27]	*Lifetime implications*: There is a 5%–15% absolute risk of developing epithelial ovarian cancer. *Clinical guidance*: Current recommendations are for a risk‐reducing salpingo‐oophorectomy at ages 45 to 50 years old. At this time, there is insufficient data to recommend screening for breast cancer, and thus, it should be guided by family history.	<1 in 500	Definite
Fumarase deficiency (FH) [105, 106]	*Lifetime implication*: Hereditary Leiomyomatosis and Renal Cell Cancer (HLRCC) is associated with a susceptibility to the development of cutaneous leiomyomas, early onset multiple uterine leiomyomas, and an aggressive form of type 2 papillary renal cell cancer. There is a 15% lifetime risk of renal malignancy. *Clinical guidance*: Recommend yearly abdominal MRI starting at age 8 to 10 years old.	<1 in 500 Ashkenazi Jewish: 1 in 99	Definite
Gaucher disease (GBA) [107, 108, 109, 110]	*Lifetime implications*: Some subtypes of GBA carriers have an increased risk of Parkinson's disease, which can occur 3–6 years earlier than noncarriers. The age‐related risk of Parkinson's disease is 1.5% at 60 years of age and 7.7% at 80 years of age, compared to 0.7% and 2.1% for noncarriers.	1 in 77 African/African American: 1 in 35 Ashkenazi Jewish: 1 in 15	Definite
GCH1‐related conditions (GCH1)^a^ [111, 112]	*Lifetime implications*: Carriers can present with GTPCH1‐deficient Dopa‐responsive dystonia or adult‐onset Parkinsonism. There is sex‐specific reduced penetrance (females have higher penetrance), so the phenotype cannot be predicted. *Clinical guidance*: Levodopa (L‐DOPA) can be used to treat symptomatic heterozygotes.	<1 in 500	Definite
Glanzmann thrombasthenia (ITGA2B, ITGB3) [113, 114, 115]	*Lifetime implications*: Heterozygotes in these genes may experience a platelet‐type bleeding disorder of altered platelet production, specifically congenital macrothrombocytopenia associated with platelet anisocytosis. *Clinical guidance*: Heterozygotes may benefit from a consultation with a hematologist. For uncontrolled bleeding, anti‐fibrinolytics, recombinant activated clotting factor VII, or platelet transfusion is the standard of care. *Pregnancy concerns*: For symptomatic heterozygotes, recombinant activated clotting factor VI or anti‐fibrinolytics are given at the time of vaginal delivery. Recombinant activated clotting factor VI is preferred at the time of cesarean delivery.	<1 in 500	Definite
Glycogen storage disease, type IB (SLC37A4) [116, 117]	*Lifetime implications*: One variant in SLC37A4 has been reported to cause congenital disorder of glycosylation type II, which is characterized by liver dysfunction, coagulation deficiencies, and profound abnormalities in N‐glycosylation of serum‐specific proteins. Ankyloglossia, cardiac abnormalities (ventricular septal defect and Tetralogy of Fallot), and scoliosis have also been reported.	1 in 158 Ashkenazi Jewish: 1 in 71	Definite
Growth hormone insensitivity syndrome (GHR) [118, 119, 120]	*Lifetime implications*: Heterozygotes in GHR may experience growth hormone insensitivity or an increased responsiveness to growth hormone, depending on the variant in GHR. In heterozygotes with growth hormone insensitivity, they may experience severe growth retardation with normal growth hormone levels but low IGF‐1/IGFBP‐3 levels and resistance to GH treatment. Heterozygous mutations have also been described to be a modifier of Familial Hypercholesterolemia in the presence of a pathogenic mutation in LDLR. *Clinical guidance*: Treatment for growth hormone insensitivity includes a combined therapy of recombinant human Growth Hormone (rhGH) with recombinant human IGF‐1.	<1 in 500	Definite
Hypophosphatasia (ALPL) [121, 122, 123]	*Lifetime* implications: Childhood‐ and adult‐onset hypophosphatasia and odontohypophosphatasia have been observed in heterozygotes. Dental abnormalities include enamel hypoplasia and premature loss of fully rooted teeth. Heterozygotes may exhibit low serum alkaline phosphatase activity and high levels of serum PLP, as well as elevated urine PEA. They are also more likely to have fractures. *Clinical guidance*: Bisphosphonates and excess vitamin D should be avoided. It is important to note that alkaline phosphatase levels increase during pregnancy, so testing antenatally may not accurately capture the clinical state of the carrier.	1 in 158 Caucasian/European: 1 in 274 Mennonite: 1 in 25	Definite
Inclusion body myopathy type 2 (GNE) [124, 125]	*Lifetime implications*: Sialuria, associated with developmental delay, coarse facial features, macroglossia, macrocephaly, and hepatomegaly, is caused by heterozygous mutations in GNE. Urinary excretion of free sialic acid is often observed in these patients.	<1 in 500 Iranian Jewish: 1 in 11	Definite
Junctional epidermolysis bullosa (COL17A1, ITGB4, LAMA3, LAMB3) [126, 127, 128, 129, 130, 131, 132]	*Lifetime implications*: Heterozygou*s* variants in COL17A1 have been associated with epidermolysis bullosa and epithelial recurrent erosion dystrophy, which is characterized by recurrent corneal erosions beginning in the first decade of life. Heterozygous variants in ITGB4 are associated with epidermolysis bullosa. These patients experience nail dystrophy of the hands and feet, acral blistering on the palms, soles, and wrists, as well as chronic granulation tissue formation in the external auditory canal. Isolated nail dystrophy has also been reported. Amelogenesis imperfecta has been reported in heterozygotes for LAMA3 and LAMB3. Their symptoms can present with pitted enamel defects.	COL17A1 and ITGB4: 1 in 500 LAMA3 and LAMB3: 1 in 781	Definite
KCNJ11‐related hyperinsulinism (KCNJ11) [133, 134, 135]	*Lifetime implications*: Heterozygotes are at risk of developing diabetes mellitus type II or permanent neonatal diabetes. At least one case has reported a heterozygous variant in KCNJ11 to be associated with developmental delay, epilepsy, and neonatal diabetes (DEND) syndrome. *Clinical guidance*: Early diabetes screening is recommended. If there is concern for congenital hyperinsulinism, then the patient should be referred to endocrinology for diabetic management with insulin.	1 in 423 Caucasian/European: 1 in 232	Definite
Leber Congenital Amaurosis (GUCY2D, RDH12, RPGRIP1) [136, 137, 138, 139, 140, 141, 142]	*Lifetime implications*: Certain carriers of Leber Congenital Amaurosis may experience vision abnormalities. GUCY2D heterozygotes may experience symptoms of cone rod dystrophy, including reduced visual acuity, photophobia, and color vision abnormality. Nearly a third have been reported to be legally blind or severely visually impaired by the fourth decade of life. Heterozygotes in RDH12 are at risk of developing retinitis pigmentosa, often with a late onset or relatively mild case when compared to homozygotes. Heterozygous RPGRIP1 variants are associated with primary open‐angle glaucoma (POAG), with varying onset (late juvenile to adult). Clinical manifestations include abnormal ERF amplitude with reduced responses to light and dark.	<1 in 500 RDH12: Caucasian/European: 1 in 456	Definite
Lethal congenital contractural syndrome 3 (PIP5K1C) [143]	*Lifetime implications*: In some children who are heterozygous for this condition, they present with microcephaly, dysmorphic features, developmental delay, seizures, and intellectual disability.	<1 in 500	Definite
Leydig cell hypoplasia (LHCGR) [144, 145]	*Lifetime implications*: Certain heterozygous variants in LHCGR have been associated with male‐limited precocious puberty. *Clinical guidance*: Treatment typically includes early intervention with spironolactone, letrozole, and Gonadotropin‐releasing hormone agonist.	<1 in 500	Definite
Limb girdle muscular dystrophy (ANO5, CAPN3, DYSF) [146, 147, 148, 149, 150, 151]	*Lifetime implications*: Several individuals with heterozygous variants in ANO5 have presented with gnathodiaphyseal dysplasia, which is characterized by bone fragility, sclerosis of tubular bones, and cemento‐osseous lesions of the jawbone. There is an autosomal dominant form of CAPN3‐related muscular dystrophy. Therefore, heterozygotes are at risk of developing symptoms, such as progressive proximal and axial muscular weakness in young adulthood. They may also experience waddling gait and scapular winging, as well as hyperkalemia. Heterozygotes for certain variants in DYSF have also been described to have hyperkalemia and late‐onset muscular dystrophy.	<1 in 500 CAPN3: Caucasian/European: 1 in 103 DYSF: Japanese: 1 in 332 Libyan Jewish: 1 in 18	Definite
Mevalonate kinase deficiency (MVK) [152, 153]	*Lifetime implications*: Certain pathogenic variants in MVK are associated with autosomal dominant disseminated superficial actinic porokeratosis. The presence of keratotic patches with elevated borders and a histological cornoid lamella characterizes porokeratosis.	<1 in 500	Definite
Mitochondrial complex IV deficiency (SCO2) [154, 155]	*Lifetime implications*: Pathogenic variants in SCO2 are associated with nonsyndromic high‐grade myopia. The mean dioptric spherical value was −22.00 in this population.	1 in 150	Definite
Mitochondrial membrane protein‐ associated neurodegeneration (c19orf12) [156, 157, 158, 159]	*Lifetime implications*: Heterozygotes may experience neurodegeneration with brain iron accumulation. These individuals experience progressive spastic paraplegia, Parkinsonism unresponsive to L‐dopa treatment, and psychiatric symptoms. They may also experience speech difficulty, optic atrophy, abnormal eye movements, dystonia, dysphagia, dysarthria, and motor axonal neuropathy.	<1 in 500	Definite
Mucopolysaccharidosis type IIIB (NAGLU) [160]	*Lifetime implications*: Autosomal Dominant Charcot–Marie–Tooth disease is associated with pathogenic variants in NAGLU. These individuals often experience recurrent leg pain, which can progress to paraesthesias in the feet and hands. Some heterozygotes will develop a sensory ataxia.	<1 in 500 Caucasian/European: 1 in 346 East Asian: 1 in 298	Definite
MYO7A‐related disorders (MYO7A) [161, 162]	*Lifetime implications*: Some pathogenic variants in MYO7A are associated with autosomal dominant hearing loss. Affected individuals may experience bilateral high‐ or low‐frequency hearing loss, but all frequencies are expected to be affected as the individual ages. Age of onset can vary between the first and fifth decades of life.	1 in 206 East Asian: 1 in 62	Definite
Neonatal hyperparathyroidism (CASR) [163, 164, 165, 166, 167]	*Lifetime implications*: Carriers of pathogenic CASR variants are at risk for hypocalcemia. Characteristics resembling Bartter syndrome, which is characterized by deficient renal reabsorption of sodium and chloride and hypokalemic metabolic alkalosis with hyperreninemia and hyperaldosteronemia, have been observed in heterozygotes as well. *Clinical guidance*: If hypercalcemia is noted, a 24‐hour urine calcium and creatinine should be performed. If urine calcium levels are low or low‐normal, familial hypocalciuric hypercalcemia should be suspected.	<1 in 500	Definite
Nephrogenic diabetes insipidus (AQP2) [168, 169, 170, 171]	*Lifetime implications*: Certain pathogenic variants in AQP2 are associated with autosomal dominant nephrogenic diabetes insipidus.	<1 in 500 Finnish population: 1 in 169	Definite
Nonsyndromic Hearing Loss (GJB6) [172, 173, 174, 175]	*Lifetime implications*: Heterozygotes may experience isolated bilateral middle/high‐frequency hearing loss or deafness. Heterozygotes have also been described to have Clouston Syndrome, a form of hidrotic ectodermal dysplasia, associated with partial‐to‐complete alopecia, nail dystrophy, and palmoplantar hyperkeratosis. Sweating is preserved and there are typically no dental anomalies.	1 in 423	Definite
Nonsyndromic Hearing Loss 7 (TMC1) [176, 177, 178]	*Lifetime implications*: Some heterozygotes may have early‐onset, progressive hearing loss.	<1 in 500	Definite
Normophosphatemic Familial Tumoral Calcinosis (SAMD9) [179, 180, 181]	*Lifetime implications*: Heterozygous carriers may have MIRAGE syndrome, which stands for myelodysplasia, infection, restriction of growth, adrenal hypoplasia, genital phenotypes, and enteropathy.	<1 in 500 Yemeni Jewish: 1 in 25	Definite
Peroxisomal acyl‐CoA oxidase deficiency (ACOX1) [182]	*Lifetime implications*: Some variants are associated with loss of glial cells, Schwann cells, and neurons.	<1 in 500	Definite
POLG‐related disorders (POLG) [183, 184, 185]	*Lifetime implications*: Heterozygotes may have progressive external ophthalmoplegia, ataxia, neuropathy, myopathy, or seizures. *Clinical guidance*: In carriers with seizure disorders, valproic acid should be avoided since it can lead to liver failure.	1 in 113	Definite
Progressive familial intrahepatic cholestasis (ABCB11, ABCB4) [186, 187, 188, 189]	*Pregnancy concerns*: Five percent of patients with intrahepatic cholestasis of pregnancy have a genetic predisposition. Heterozygotes should be monitored for the development of intrahepatic cholestasis of pregnancy. *Lifetime implications*: Increased risk for intrahepatic cholestasis of pregnancy, cirrhosis, cholelithiasis, colangiocarcinoma, or hepatocellular carcinoma. *Clinical Guidance*: Monitor for the development of hepatic disease.	1 in 112 <1 in 500	Definite
Progressive myoclonic epilepsy, type 1B (PRICKLE1) [190, 191, 192, 193, 194]	*Lifetime implications*: There are reports of carriers having non‐myoclonic seizures, myoclonic seizures, developmental delay, mild intellectual disability, autism, distal polyneuropathy, or central nervous system malformations.	<1 in 500	Definite
Prothrombin (F2) [87, 195, 196, 197, 198, 199, 200]	*Pregnancy considerations*: The risk of venous thrombotic embolism (VTE) during pregnancy ranges from <1% to 10%, depending on family and personal history. There is a 3x–15x increased risk of VTE during pregnancy. *Lifetime risk*: Risk of VTE is approximately 1% per year. *Clinical guidance*: During pregnancy, if there is no personal or family history of VTE, prophylactic anticoagulation can be considered postpartum. If there is a first‐degree relative with VTE but no personal history, antepartum anticoagulation can be considered, and postpartum prophylactic anticoagulation is recommended. If there is a history of VTE, then anticoagulation is recommended antepartum and postpartum. Combined oral contraceptives should be avoided.	1 in 33	Definite
Retinitis pigmentosa 20 (RPE65)^a^ [201, 202, 203]	*Lifetime implications*: In some variants, carriers may present with retinal dystrophy with choroidal involvement.	1 in 228	Definite
Retinitis pigmentosa 37 (NR2E3) [142, 204]	*Lifetime implications*: Some variants are associated with retinal dystrophy.	1 in 209	Definite
Retinitis pigmentosa 47 (SAG)^a^ [142, 205]	*Lifetime implications*: Some carriers present with symptoms of retinitis pigmentosa.	1 in 228	Definite
Retinitis pigmentosa 59 (DHDDS) [206, 207]	*Lifetime implications*: Developmental and epileptic encephalopathy has been reported with some variants of this gene.	1 in 296 Ashkenazi Jewish: 1 in 118	Definite
Rh Deficiency syndrome (RHAG) [208, 209]	*Lifetime implications*: Carriers may develop hemolytic anemia due to defective iron channels in red blood cells, which can lead to overhydration and hereditary stomatocytosis.	<1 in 500	Definite
RYR1‐related conditions (RYR1) [210, 211, 212, 213, 214, 215]	*Lifetime implications*: The most common phenotypes include King‐Denborough syndrome (characterized by skeletal deformities, short stature, joint contractures, and pectus carinatum), central core disease (manifesting as hypotonia and motor development delay), and malignant hyperthermia. *Clinical guidance*: The diagnosis is confirmed on a muscle biopsy. Symptoms can be treated with off‐label use of N‐acetylcysteine, dantrolene, or salbutamol/albuterol. Heterozygotes should be aware of the risk of malignant hyperthermia and avoid exposure to inhalational anesthetics or suxamethonium.	<1 in 500	Definite
Sepiapterin reductase deficiency (SPR) [216, 217, 218, 219]	*Lifetime implications*: Several reports suggest the possibility of manifesting carriers. There is one report of DOPA‐responsive dystonia and another case report of childhood‐onset Parkinson's disease. There is also a case series of familial carrier status segregated by fibromyalgia.	<1 in 5,000	Definite
Spastic paraplegia type 7 (SPG7) [220, 221, 222]	*Lifetime implications*: In some variants, heterozygote carriers may present with optic neuropathy but typically do not have spasticity until adulthood.	1 in 159	Definite
Spondylometaepiphyseal dysplasia (DDR2) [223]	*Lifetime implications*: Carriers can present with contractures of the fingers, corneal neovascularization, acro‐osteolysis, subcutaneous tissue wasting, chronic skin ulcers, and keloid formation.	<1 in 500	Definite
T‐cell immunodeficiency with congenital alopecia and nail dystrophy (FOXN1) [224]	*Lifetime implications*: There have been reports of lymphopenia during infancy, often associated with nail dystrophy. Adults with this variant typically have normal CD4+ but lower than normal CD8+ cell counts.	<1 in 500	Definite
Trifunctional protein deficiency (HADHA, HADHB) [225, 226, 227, 228, 229, 230, 231, 232]	*Pregnancy concerns*: If the fetus is affected by trifunctional protein deficiency, there is a 15%–62% maternal risk of HELLP syndrome or acute fatty liver of pregnancy. The mechanism of this increased risk is poorly understood but hypothesized to be due to either maternal heterozygosity leading to hepatic insufficiency or fetal overproduction of hydroxyaryl derivatives. *Clinical guidance*: Heterozygotes who are carriers for this condition should be monitored for HELLP and AFLP during pregnancy with monthly liver function testing until the third trimester, when it should be performed more frequently. Multidisciplinary management with MFM and/or a biomedical geneticist.	<1 in 500 Finnish: 1 in 124	Definite
Usher syndrome, type IC (USH1C)^a^ [233]	*Lifetime implications*: Some alleles (c.667G > T [p.Gly223Cys]) have been reported to be autosomal dominant and associated with nonsyndromic hearing loss.	1 in 353 French Canadian: 1 in 227	Definite
Weyers acrodental dysostosis, EVC2‐related (EVC2) [234, 235]	*Lifetime implications*: Heterozygous carriers with specific variants (especially EVC2 truncating variants in exon 22) have been reported to manifest a milder form of Weyers acrofacial dysostosis.	1 in 240 Amish: 1 in 7	Definite
Zellweger syndrome, PEX6‐related (PEX6) [236, 237]	*Lifetime implications*: Depending on the allele, there are reports of one PEX6 variant, p.Arg860Trp, being associated with Zellweger syndrome due to allelic imbalance. The presentation is typically milder than that with homozygous PEX6 mutations.	1 in 280 Yemenite Jewish: 1 in 18	Definite

^a^ Variants can be either autosomal dominant or autosomal recessive.

^b^ Autosomal dominant.

### 2.2 X‐linked conditions with clinical implications in the carrier state

X‐linked disorders often affect females, depending on the degree of X inactivation, also called lyonization. The degree of lyonization cannot be predicted, as it can vary across organ systems. Thirty‐eight X‐linked disorders are described as having definite implications and are summarized in Table 2. One of the most common X‐linked disorders with implications for female carriers is Fragile X. Female carriers of both premutations and full mutations are at increased risk for fragile X–associated neuropsychiatric disorders, primary ovarian insufficiency, and, in rare cases, tremor/ataxia syndrome [238, 239, 240, 241, 242, 243, 244].

**TABLE 2:** X‐linked genes with definitive evidence of causing risks for the carrier in the heterozygote state. Carrier frequency is described for the general population except where a specific ethnicity is described.

Condition (gene)	Pregnancy concerns/risks for female heterozygotes/clinical guidance	Carrier frequency (ethnicity) [13, 17]	Supportive evidence level [16]
Adrenoleukodystrophy, X‐linked (ABCD1) [245, 246, 247]	*Risk for female heterozygotes*: Most females are asymptomatic in childhood. In adulthood, 20%–80% of female carriers will develop mild to moderate spastic paraparesis with bladder and bowel issues, abnormal gait, hypertonia, and urinary symptoms. *Clinical guidance*: Symptomatic females can receive multidisciplinary supportive treatment.	1 in 21,000	Definite
ALG13‐Related Conditions (ALG13) [248, 249, 250, 251]	*Risk for female heterozygotes*: A case series has identified some females with early‐onset severe epilepsy and developmental delay.	<1 in 50,000	Definite
Allan‐Herndon‐Dudley syndrome (SLC16A2) [252, 253, 254]	*Risks for female heterozygotes*: Females can have hypothyroidism. There are case reports of females with developmental delay and intellectual disability. *Clinical guidance*: Thyroid function should be evaluated.	<1 in 500	Definite
Alpha thalassemia X‐linked intellectual disability syndrome (ATRX) [255, 256, 257]	*Risks for female heterozygotes*: Most females have skewed X inactivation of the ATRX variant. There are select case reports of females with intellectual differences.	<1 in 250,000	Definite
Alport syndrome, COL4A5‐related (COL4A3, COL4A4, COL4A5) [258, 259, 260]	*Risks for female heterozygotes*: Approximately 95% of females exhibit persistent or intermittent microhematuria and proteinuria, which develops in approximately 75%. *Pregnancy concerns*: Increased risk of complications, including increased preeclampsia, renal insufficiency, proteinuria, or worsened hypertension. *Clinical guidance*: Carriers should be screened every one to two years for microhematuria and microalbuminuria, and undergo annual blood pressure monitoring. If microalbuminuria is present, renin‐angiotensin‐aldosterone system blockers should be initiated to delay the progression to end‐stage renal disease. However, in female heterozygotes with renal disease, other etiologies of renal disease should also be excluded. Hearing aids may be needed for those with hearing loss. During pregnancy, the patient should be monitored for signs and symptoms of preeclampsia.	1 in 139	Definite
Androgen insensitivity syndrome (AR) [261, 262]	*Risks for female heterozygotes*: Random X inactivation can lead to asymmetrical distribution of pubic and axillary hair in 10% of female carriers.	1 in 14,286	Definite
Antley‐Bixler syndrome (POR) [263]	*Risks for female heterozygotes*: Carriers may have altered hepatic drug metabolism. *Clinical guidance*: Caution should be used with any drugs metabolized by the liver.	1 in 159	Definite
Charcot–Marie–Tooth disease, X‐linked type 1 (GJB1) [264, 265, 266]	*Risks for female heterozygotes*: Females may have mild symptoms of peripheral neuropathy that can progress to moderate. There are a few reports of females with stroke‐like episodes.	1 in 667	Definite
Creatine deficiency syndrome (SLC6A8) [267, 268]	*Risks for female heterozygotes*: Female carriers have been reported to have learning difficulties, mild intellectual delay, behavioral problems, or seizures.	1 in 3,434	Definite
Dystrophinopathies (Duchenne and Becker's Muscular Dystrophies) (DMD) [8, 9, 10, 11]	*Risks for female heterozygotes*: Female heterozygous carriers can develop later onset mild dilated cardiomyopathy (3%–33%), usually around age 40‐50s, typically with slower progression of disease. Muscle weakness (14‐19%) and myalgia (5%) have been reported. They also usually have increased serum creatine kinase (2x‐10x normal). *Clinical guidance*: Female heterozygotes should undergo cardiac evaluation every five years starting at age 25.	1 in 2,350	Definite
Emery‐Dreifuss muscular dystrophy (EMD) [269, 270, 271, 272, 273]	*Risks for female heterozygotes*: Female heterozygotes are usually asymptomatic but have been reported to be at increased risk of developing the disease, other cardiac diseases (including sudden death), or progressive muscular dystrophy. Late‐onset cardiac disease, typically after the fifth decade of life, has been reported. *Clinical guidance*: Cardiac evaluation is recommended for individuals who are carriers.	1 in 81,967	Definite
Fabry disease (GLA) [274, 275, 276, 277]	*Risk for female heterozygotes*: Heterozygous females may present with a wide range of symptoms, from severe to completely asymptomatic. Findings include angiokeratomas (10%‐50%), acroparesthesia (50%‐90%), cornea verticillate (70‐90%), renal failure (10%), increased risk for moderate left ventricular hypertrophy, and some reports of psychiatric disease. Obstructive lung disease is seen more commonly in smokers. *Clinical guidance*: Renal and cardiac function should be assessed regularly. Smoking cessation is strongly advised. Enzyme replacement therapy can be considered if there is histological, laboratory, or imaging evidence of nervous, heart, or kidney disease. *Pregnancy concerns*: During pregnancy, heterozygotes should also be monitored for depression. There is an increased risk for intrapartum headaches, acroparesthesias, proteinuria, hypertension, gastrointestinal symptoms, and postpartum depression.	1 in 25,000	Definite
FHL1‐related neuromuscular disorders (FHL1) [269, 278]	*Risks for female heterozygotes*: Heterozygous females are usually asymptomatic but are at risk of developing progressive muscular dystrophy, cardiac diseases, or Emery‐Dreifuss Muscular Dystrophy phenotype. *Clinical guidance*: Cardiac evaluation is recommended for individuals who are carriers.	<1 in 50,000	Definite
Fragile X syndrome (FMR1) [238, 239, 240, 241, 242, 243, 244]	*Risks for female heterozygotes*: Females who are heterozygous for a premutation are at increased risk for fragile X‐associated neuropsychiatric disorders, tremor/ataxia syndrome, and primary ovarian insufficiency (20‐30%). Full mutation females are at risk for presenting with some of the features of Fragile X, including seizures (3%–7%), autism (20%), 70% will have borderline to normal IQ ranges, but with learning differences, anxiety disorders are commonly reported (77%), and fragile X‐associated tremor/ataxia syndrome (16%). Females with fragile X‐associated tremor/ataxia often also present with hypothyroidism (50%), peripheral neuropathy (53%), or muscle pain (73%). If the allele is <55 repeats (intermediate range): not a carrier, offspring not at risk for *FMR1* disorder. If the allele is between 55 and 200 (premutation allele), offspring are at risk of inheriting the disease. If an allele with more than 200 repeats (full mutation range) is a carrier, the offspring will have a 50% chance of inheriting the disease. *Clinical guidance*: Female carriers may have diminished ovarian reserve and can experience early menopause. Fertility preservation can be considered.	1 in 259 Ashkenazi Jewish: 1 in 115	Definite
Glucose‐6‐phosphate dehydrogenase deficiency (G6PD) [279, 280, 281, 282]	*Risks for female heterozygotes*: Due to random X‐inactivation, female heterozygotes may have low G6PD activity in their erythrocytes. Hemolysis with need for transfusion has been reported with high doses of primaquine and favism. There is also an increased risk for neonatal hyperbilirubinemia. *Clinical guidance*: During pregnancy and lactation, carriers should avoid pharmacological or chemical exposures that are known to be unsafe in individuals with G6PD deficiency. *Pregnancy concerns*: Based on expert opinion, during pregnancy and lactation, carriers should avoid pharmacologic or chemical exposures that are known to be unsafe for G6PD.	1 in 7	Definite
Hemophilia A (F8) [283, 284]	*Risk for female heterozygotes*: Regardless of the familial phenotype, female carriers may experience a failure to increase factor VIII clotting activity in response to stress. Nearly a third of females will have factor VIII clotting activity low enough to increase bleeding risk. An additional quarter of females report increased bleeding tendencies even with normal factor VIII clotting activity levels. *Pregnancy concerns*: Affected male fetuses may have intracranial hemorrhage. There are conflicting recommendations for cesarean versus vaginal delivery. However, operative vaginal delivery should be avoided. *Clinical guidance*: Factor VIII clotting activity should be assessed at the initial prenatal visit, 28 weeks, 34 weeks, and before delivery. The levels typically rise during pregnancy, but optimal levels have not been established. It is typically recommended that target levels be greater than 50% for neuraxial anesthesia and greater than 100% for delivery. There is an increased risk for postpartum hemorrhage due to the postpartum decrease in Factor VIII clotting activity. An anti‐fibrinolytic agent should be given immediately after delivery and then daily for two weeks.	1 in 3,250	Definite
Hemophilia B (F9) [284, 285]	*Risk for female heterozygotes*: Bleeding risk for female heterozygotes depends on factor IX clotting activity. The high risk is for those with levels less than 40%, which occurs in approximately 30% of female carriers. *Pregnancy concerns*: Factor IX levels do not rise in pregnancy, and carriers often require factor IX infusions during delivery. An anti‐fibrinolytic agent should be given immediately after delivery and then daily for two weeks. If there is an affected male fetus, shared decision‐making should be made for the mode of delivery. *Clinical guidance*: Factor XI clotting activity should be assessed at the initial prenatal visit, 28 weeks, 34 weeks, and prior to delivery. Anti‐fibrinolytic agents may be needed during surgery or delivery.	1 in 15,000	Definite
HSD10 mitochondrial disease (HSD17B10) [286, 287, 288]	*Risks for female heterozygotes*: A heterozygous female may experience developmental delay and intellectual delay. These symptoms are typically not progressive. However, infantile onset HSD10 disease with delayed neurologic development and/or regression has been described in heterozygous females due to skewed X‐inactivation. *Clinical guidance*: Symptomatic carriers may be treated with an isoleucine‐restricted diet.	<1 in 50,000	Definite
LAMP‐2 deficiency (LAMP2) [289, 290, 291, 292]	*Risk for female heterozygotes*: Approximately one‐third of female heterozygote carriers may experience muscular weakness and atrophy, which is typically central but can also affect distal muscles. Nearly two‐thirds of females will also have elevated serum kinase. Cognitive impairment and learning differences have been reported in 6%–47%. Females frequently get hypertrophic cardiomyopathy, but it often presents later than their male counterparts. *Clinical guidance*: Female heterozygotes should undergo cardiac evaluation in the first year of life. Starting at age 6, they should undergo annual multidisciplinary follow‐up, which includes laboratory tests, an ECG, an echocardiogram, and 24‐hour Holter monitoring.	Rare but the exact frequency is unknown.	Definite
Lowe syndrome (OCRL) [293, 294, 295, 296]	*Risks for female heterozygotes*: Characteristic lens opacities are seen on slit lamp examination for 95% of postpubertal females. It is estimated that approximately 10% will develop cataracts. Additionally, some females may experience hypercalcinuria, renal calculi, or moderate low‐molecular‐weight proteinuria.	1 in 250,000	Definite
Mecp2‐related conditions (MECP2) [297, 298, 299, 300, 301, 302]	*Risks for female heterozygotes*: Females can present with classic Rett syndrome, characterized by normal development during the first 6 to 18 months of life, followed by rapid regression. Some females are less impacted, with only mild learning differences, which can be due to skewed X inactivation. Periventricular cystic lesions have been reported.	<1 in 10,000	Definite
Mucopolysaccharidosis type II (IDS) [303, 304, 305, 306]	*Risks for female heterozygotes*: Approximately 10‐20% of heterozygous female individuals may exhibit findings due to X‐inactivation. Females may experience skeletal anomalies, liver abnormalities, carpal tunnel syndrome, recurrent ear infection, hypoacusia, or odontological problems without coarse facial features. They may experience muscle weakness, fatigue, muscle pain, and delayed psychomotor and speech development. Symptoms resembling those expected in affected males have been reported in heterozygote females. *Clinical guidance*: Enzyme replacement therapy has been used to stabilize the disease in females with manifestations.	1 in 50,000	Definite
Nephrogenic diabetes insipidus (AVPR2) [168, 169, 170, 171]	*Risks for female heterozygotes*: Certain pathogenic variants in AQP2 are associated with autosomal dominant nephrogenic diabetes insipidus. Due to X‐inactivation, carriers of AVPR2 may also exhibit symptoms of nephrogenic diabetes insipidus. Polyuria and polydipsia have been observed in heterozygous females.	<1 in 50,000	Definite
Ornithine transcarbamylase deficiency (OTC) [307, 308, 309]	*Pregnancy concerns*: Hyperammonia can develop in the peripartum or postpartum periods which can lead to life‐threatening catabolic episodes in the peripartum period. *Risks for female heterozygotes*: Carriers can experience hypoglycemia when fasting. Encephalopathic episodes occur in 18% of female heterozygotes. *Clinical guidance*: Multidisciplinary management during delivery and postpartum is necessary to monitor and treat elevated ammonia levels. Treatment can include adjusting the diet, monitoring ammonia levels, administering dextrose infusions, and using sodium phenylbutyrate, arginine HCl, or a combination of sodium phenylacetate and sodium benzoate.	1 in 5,000	Definite
Phosphoglycerate kinase 1 deficiency (PGK1) [310, 311, 312]	*Risks for female heterozygotes*: There have been reports of hemolytic anemia in female heterozygotes.	<1 in 50,000	Definite
PRPS1‐related disorders (PRPS1) [313, 314, 315, 316, 317, 318]	*Risks for female heterozygotes*: Optic atrophy and retinal dystrophy are the most common findings. There is a variable presentation with ataxia, progressive peripheral neuropathy, and hearing loss. Charcot–Marie–Tooth disease, gout, and Arts syndrome have been reported. *Clinical guidance*: There have been small trials of *S*‐adenosylmethionine, which have shown improvement in symptoms.	<1 in 250,000	Definite
Pyruvate dehydrogenase E1‐alpha deficiency (PDHA1) [319, 320, 321, 322]	*Risks for female heterozygotes*: Females with a pathogenic variant resulting from an insertion or deletion can be affected. However, females with a missense pathogenic variant tend to be asymptomatic. There are reports of females with significant findings on imaging, peripheral neuropathy, or facial stigmata. *Clinical guidance*: Affected females should follow a ketogenic diet.	<1 in 250,000	Definite
RPS6KA3‐Related Intellectual Disability [323, 324]	*Risks for female heterozygotes*: Developmental delay with or without intellectual disability is the most common presentation in females. They may also have some mild physical features, such as facial coarsening or tapered fingers. Nearly a third have progressive kyphoscoliosis.	<1 in 50,000	Definite
Spastic paraplegia type 2 (PLP1) [325, 326, 327, 328]	*Risks for female heterozygotes*: Approximately 20% of females are affected with nystagmus, hypotonia, cognitive impairment, psychosis, mild peripheral neuropathy, or progressive lower limb spasticity. If the familial variant presents as severe in males, then females tend to be unaffected. It is hypothesized that severely impacted oligodendrocytes in females undergo degeneration and are replaced by those that have a functioning gene, resulting in skewed X inactivation toward the functional gene.	<1 in 50,000	Definite
X‐linked central hypothyroidism and testicular enlargement (IGSF1) [329]	*Risk for female heterozygotes*: One quarter of female carriers have central hypothyroidism. *Pregnancy concerns*: Although there are no published guidelines, it is reasonable to monitor thyroid function during pregnancy.	<1 in 50,000	Definite
X‐linked dyskeratosis congenita (DKC1) [330, 331]	*Pregnancy concerns*: In females with skewed X inactivation, they may become pancytopenic. *Risks for female heterozygotes*: Female carriers may experience skin pigmentation differences, bone marrow failure, or nail dysplasia. *Clinical guidance*: A complete blood count (CBC) should be performed to evaluate for pancytopenia. The frequency of this monitoring has not been established and should be guided by the patient's history.	<1 in 50,000	Definite
X‐linked epilepsy with variable learning disabilities (SYN1) [332, 333, 334]	*Risks for female heterozygotes*: Some female carriers can present with psychiatric disorders or febrile seizures.	<1 in 50,000	Definite
X‐linked hearing loss, POU3F4‐related (POU3F4) [316, 335, 336]	*Risks for female heterozygotes*: Female carriers can have mild hearing differences.	<1 in 50,000	Definite
X‐linked intellectual disability with cerebellar hypoplasia and distinctive facial appearance (OPHN1) [337, 338, 339]	*Risks for female heterozygotes*: Depending on X inactivation, some females may have mild intellectual differences, seizures, and mild cerebellar hypoplasia.	<1 in 50,000	Definite
X‐linked intellectual disability, ARX‐related (ARX) [340, 341]	*Risks for female heterozygotes*: 43% asymptomatic, 16% have isolated agenesis of the corpus callosum or mild intellectual differences, 41% have severe developmental delays or epileptic encephalopathy.	<1 in 50,000	Definite
X‐linked intellectual disability, DLG3‐related (DLG3) [342, 343]	*Risks for female heterozygotes*: There are reports of intellectual differences in a female carrier.	<1 in 50,000	Definite
X‐linked intellectual disability, KDM5C‐related (KDM5C) [344, 345]	*Risks for female heterozygotes*: Reports have been made of intellectual and developmental differences, as well as a narrow palate.	<1 in 50,000	Definite
X‐linked retinitis pigmentosa, RPGR related (RPGR) [142, 346, 347, 348, 349, 350, 351, 352]	*Risks for female heterozygotes*: Depending on X‐inactivation, females can be unaffected or express mild to severe ocular disorders, most commonly retinal pigmentary changes. In addition, there are reports of infertility due to primary ciliary dyskinesia. *Clinical guidance*: Female carriers with infertility may benefit from assisted reproductive technology.	1 in 3,000	Definite

For most X‐linked conditions, predicting a phenotype in a female carrier is challenging. However, some variants have a known pattern of X inactivation. For example, alpha thalassemia X‐linked intellectual disability syndrome (ATRX) [255, 256] tends to have skewed X inactivation of the variant, so most females do not present with a phenotype. Spastic paraplegia type 2 (PLP1) tends to have skewed X inactivation toward the functional gene in those with severe familial variants, and females are more often affected if they have a mild familial variant [325, 326, 327, 328].

### 2.3 Variants without strong supporting evidence

Additional conditions with possible manifestations in carriers are included in Tables 3 (autosomal recessive conditions) and Table 4 (X‐linked conditions). The conditions in these tables have moderate or limited evidence. Moderate evidence describes genes with several probands with conditions supporting disease causality and moderate experimental data supporting gene‐disease association. Whereas limited describes genes with fewer than three observations, or probands lack sufficient evidence for disease causality. [16] It should be acknowledged that, over time, these genes may change to become strong or definitive, or, conversely, may have contradictory evidence. It should also be noted that many X‐linked conditions are rare; although implications for female heterozygotes may not have been previously reported, carriers may still exhibit manifestations due to skewed X‐inactivation.

**TABLE 3:** Genes that have moderate or limited evidence of causing risk for the carrier in the heterozygote state. All genes are autosomal recessive, except where it is specified that there are autosomal dominant forms for the gene. Carrier frequency is described for the general population except where a specific ethnicity is described.

Condition (gene)	Pregnancy concerns/lifetime implications/clinical guidance	Carrier frequency (ethnicity) [13, 17]	Supportive evidence level [16]
17‐Beta hydroxysteroid dehydrogenase (HSD17B3) [353]	*Pregnancy concerns*: One study showed that decreased 17‐beta‐hydrosteroid dehydrogenase type 1 levels in the second trimester were an independent risk factor predicting preeclampsia in pregnancy.	1 in 192 Palestinian: 1 in 8	Limited
Aceruloplasminemia (CP) [354, 355]	*Lifetime implications*: There are reports of heterozygous carriers who presented with cerebellar signs or movement disorders. *Clinical guidance*: Symptomatic heterozygotes are typically treated with iron chelation agents.	<1 in 500	Moderate
Aromatase deficiency (CYP19A1) [356, 357, 358]	*Pregnancy concerns*: Although studies have shown that there are lower levels of aromatase in pregnancies affected with preeclampsia, to date, there have been no studies implicating that carriers are at an increased risk of preeclampsia. If the fetus is affected by the disorder, it is typical to see hirsutism and virtualization in the pregnant female.	<1 in 500	Limited
Beta thalassemia (HBB) [359, 360]	*Lifetime implications*: Most carriers are asymptomatic but can have microcytic mild anemia or microcytosis without anemia. *Clinical guidance*: Hemoglobin with mean corpuscular volume should be evaluated.	1 in 158 African/African American: 1 in 10 East Asian 1 in 50 Latino: 1 in 128 Mediterranean: 1 in 3 South Asian/Indian: 1 in 25	Moderate
Bloom syndrome (BLM) [361, 362]	*Lifetime implications*: Studies have identified a higher rate of BLM heterozygotes in people with endometrial cancer, mesothelioma, and colorectal cancer. *Clinical guidance*: There are no current National Comprehensive Cancer Network (NCCN) guidelines. A detailed family history should be obtained.	1 in 800 Ashkenazi Jewish: 1 in 134	Limited
Carnitine palmitoyltransferase II deficiency (CPT2) [363, 364]	*Lifetime implications*: Carriers may have impaired fat oxidation during exercise and may experience myopathic symptoms.	<1 in 500 Ashkenazi Jewish: 1 in 51	Moderate
Catecholaminergic polymorphic ventricular tachycardia (CASQ2) [365, 366, 367, 368]	*Lifetime implications*: Heterozygotes can have exercise‐ or emotion‐induced bidirectional or polymorphic ventricular tachycardia. *Clinical guidance*: Cardiac evaluation and, if symptomatic, nadolol or another non‐selective beta blocker should be used.	1 in 224	Moderate
Congenital adrenal hyperplasia due to 21‐hydroxylase deficiency (CYP21A2) [369]	*Lifetime implications*: There is some limited evidence that carriers may be at increased risk for polycystic ovarian syndrome.	1 in 61 Inuit: 1 in 9 Middle‐Eastern: 1 in 35	Limited
Congenital amegakaryocytic thrombocytopenia (MPL) [370, 371, 372]	*Lifetime implications*: Depending on the variant, carriers can have ringed sideroblasts with thrombocytosis or acute myeloid leukemia. In other variants, thrombocytopenia has been reported. *Clinical guidance*: For symptomatic heterozygotes, they may require transfusion, stem cell transplantation, or pharmacotherapy.	1 in 102 Ashkenazi Jewish: 1 in 55	Moderate
Congenital hypothyroidism, DUOX2‐related (DUOX2) [373, 374, 375]	*Lifetime implications*: Carriers can present with transient or permanent hypothyroidism.	1 in 366	Moderate
Congenital myasthenic syndrome (CHRNE) [376, 377, 378]	*Lifetime implications*: This can present as congenital myasthenia gravis.	1 in 408	Moderate
Corneal endothelial dystrophy (SLC4A11) [379, 380, 381]	*Lifetime implications*: Carriers have been reported to be at increased risk of developing Fuchs endothelial dystrophy later in life.	<1 in 500	Moderate
Cortical dysplasia‐focal epilepsy syndrome (CNTNAP2) [382]	*Lifetime implications*: CNTNAP2 variant is seen in autism spectrum disorder, intellectual disability, and schizophrenia. Carrier risk has a high correlation to a specific type of variant seen in a particular family.	<1 in 500	Limited
CRB1‐related retinopathy (CRB1) [383]	*Lifetime implications*: Carriers have been reported to have regional retinal defects.	1 in 104	Moderate
Dihydropyrimidine dehydrogenase deficiency (DPYD) [384]	*Lifetime implications*: Carriers have reduced clearance of 5‐fluorouracil.	<1 in 500	Moderate
Dubin Johnson syndrome (ABCC2) [186, 385, 386]	*Pregnancy concerns*: Some studies have shown an increased risk in intrahepatic cholestasis of pregnancy.	Iranian and Moroccan Jews: 1 in 18	Moderate
Ehlers–Danlos‐like syndrome due to tenascin‐X deficiency (TNXB) [387, 388, 389]	*Lifetime implications*: Carriers have been reported to have recurring joint dislocations, generalized joint hypermobility, and chronic joint pain. *Pregnancy considerations*: Poor pregnancy outcomes (postpartum hemorrhage, uterine rupture, preterm birth) and esophageal rupture at the time of intubation have been reported in full mutation carriers. It is not known what the risk is for heterozygote carriers; however, they have been shown to have decreased TNX serum levels. Nevertheless, care should be taken during endotracheal intubation, especially when it is performed in an emergency.	1 in 28	Moderate
Fanconi Anemia, Group A (FANCA)^a^ [390]	*Lifetime implications*: There is one study that has shown an increased risk of breast and ovarian cancer in mutation carriers. *Clinical guidance*: There are no specific NCCN recommendations at this time, but it is reasonable to tailor screening based on family history.	1 in 239 Moroccan Jewish: 1 in 100 Indian Jewish: 1 in 27	Moderate
Galactokinase deficiency (GALK1) [391, 392]	*Lifetime implications*: Heterozygous carriers may have an increased incidence of cataracts later in life.	1 in 110 Irish: 1 in 64	Limited
Generalized thyrotropin‐releasing hormone resistance (TRHR) [393, 394]	*Lifetime implications*: Heterozygous mutation carriers may present with subclinical hypothyroidism, which typically does not require replacement therapy.	<1 in 500	Limited
Gitelman syndrome (SLC12A3) [395, 396]	*Lifetime implications*: Lower potassium and chloride levels have been observed in carriers. There is a report of an increase in fractures, although the mechanism is poorly understood. *Clinical guidance*: Recommend monitoring chloride and potassium levels during periods of fluid shifts or when there is a risk of electrolyte disturbances.	1 in 100	Limited
Glycogen storage disease type V (PYGM) [397, 398]	*Lifetime implications*: Heterozygotes may develop muscle symptoms that are probably related to the critically low residual level (30%–40%) of myophosphorylase activity in muscle. They may present with myalgia and weakness after exercise.	<1 in 500 Caucasian/European: 1 in 206	Moderate
Hartnup disorder (SLC6A19) [399]	*Pregnancy concerns*: Pregnant females may experience lower tryptophan concentrations when on a restricted diet of depleted vitamin B3. This results in reduced nicotinamide adenine dinucleotide levels, which are associated with an increased probability of embryo loss.	1 in 87	Limited
Hemochromatosis, type 2A (HJV) [400]	*Lifetime implications*: Heterozygotes can develop middle‐age‐onset hemochromatosis or iron overload.	1 in 500	Limited
Hemophagocytic lymphohistiocytosis, familial, 2 (PRF1) [401, 402]	*Lifetime implications*: Heterozygotes are at risk for adult‐onset aplastic anemia. Hemophagocytosis on bone marrow biopsy has been observed in several heterozygotes. The perforin protein levels were low, perforin granules were absent, and natural killer cell cytotoxicity was decreased in this population. *Clinical guidance*: It is extremely rare for heterozygotes to be symptomatic. However, if the familial variant is known to cause symptoms of aplastic anemia in the carrier state, then the proband should be monitored closely, as they may benefit from prophylactic hematopoietic stem cell transplantation, especially during febrile episodes.	1 in 149	Limited
Hereditary fructose intolerance (ALDOB) [403, 404]	*Lifetime risks*: Carriers are at risk for hyperuricemia, posing an increased risk for gout when exposed to elevated levels of fructose.	1 in 122 African/African American: 1 in 250 Caucasian/European: 1 in 67 Middle‐Eastern: 1 in 97	Limited
Hereditary Hemochromatosis (HFE) [405, 406, 407]	*Lifetime risks*: Heterozygotes for specific variants may have elevated serum transferrin saturation and serum ferritin concentrations. Heterozygotes will rarely experience complications of iron overload. *Clinical guidance*: Evaluation of serum ferritin and transferrin saturation is recommended. For individuals with elevated serum ferritin levels, phlebotomy therapy is recommended.	1 in 10 African/African American: 1 in 17 Caucasian/European: 1 in 3 East Asian: 1 in 12 Latino: 1 in 6	Limited
Hereditary hypophosphatemic rickets with hypercalciuria (SLC34A3) [408]	*Lifetime risks*: Heterozygous individuals may have hypercalciuria with mild hypophosphatemia and/or elevations in 1,25‐dihydroxyvitamin D levels.	<1 in 500	Limited
Holocarboxylase synthetase deficiency (HLCS) [409]	*Lifetime risks*: The plasma total homocysteine concentration response after methionine loading was observed to be abnormal in heterozygotes.	1 in 500	Limited
IFT140‐related disorders (IFT140) [410, 411]	*Lifetime implications*: Polycystic kidney disease has been observed in heterozygotes. These patients can present with bilateral kidney cysts with possible renal failure. Cardiomyopathy has also been observed in some heterozygotes. *Clinical guidance*: In patients with rapidly progressing disease, vasopressin V2 receptor antagonists may slow the progression of renal disease. Patients with hypertension should be treated with a renin‐angiotensin‐aldosterone system blocker. The onset of end‐stage renal disease may be delayed with a renal diet, lipid control, and maintaining a normal body weight.	<1 in 500	Moderate
Immunodeficiency 15B (IKBKB) [412]	*Lifetime implications*: Heterozygote variants in IKBKB are associated with late‐onset recurrent respiratory tract infections and lymphopenia. Combined T and B cell deficiency is observed in these heterozygotes. Patients may also experience eczema, ectodermal dysplasia, hidradenitis suppurativa, subcutaneous abscesses, mucocutaneous candidiasis, and premature cataracts.	<1 in 500	Moderate
Immunodeficiency 23 (PGM3) [413]	*Lifetime implications*: Mild Idiopathic Focal Epilepsy has been reported in heterozygotes for PGM3.	<1 in 500	Limited
Immunodeficiency with Hyper IgM syndrome (AICDA) [414]	*Lifetime implications*: Some variants are associated with hyper‐IgM syndrome.	<1 in 500	Limited
Infantile neuroaxonal dystrophy (PLA2G6) [415]	*Lifetime implications*: Heterozygous PLA2G6 variants have been observed in patients with L‐dopa‐responsive Parkinson's disease. These patients may not have dystonia. Cognitive decline may appear over time. These patients’ MRIs show iron accumulation in neo and paleostriatum, but cerebellar atrophy is absent.	1 in 500	Limited
Isolated growth hormone deficiency (GHRHR) [416]	*Lifetime implications*: Older individuals (60–80 years) have been observed to have reduced stature. This same degree of short stature was not observed in younger heterozygotes, indicating that the GHRHR can have different effects throughout the lifespan.	<1 in 500	Limited
Limb‐girdle muscular dystrophy (SGCD) [417]	*Lifetime implications*: Certain variants in the SGCD gene have been associated with autosomal dominant cardiomyopathy. This has been debated and continues to be investigated. *Clinical guidance*: Cardiac evaluation is recommended for individuals who are carriers.	<1 in 500	Moderate
Metachromatic leukodystrophy due to saposin‐b deficiency (PSAP) [418, 419, 420]	*Lifetime implications*: Certain variants in PSAP are associated with autosomal dominant Parkinson's disease. Age of onset varies, and cases of early onset (<50 years old) Parkinson's disease have been reported.	<1 in 500	Moderate
Methionine adenosyltransferase (MAT1A) [421, 422]	*Lifetime implications*: Certain variants are associated with persistent isolated hypermethioninemia in carriers. Heterozygotes can have mild methionine elevations, but this is unlikely to cause the neurological symptoms seen in homozygotes. However, heterozygotes are likely to be identified by newborn screening given the elevated methionine levels.	<1 in 500	Limited
Mitochondrial neurogastrointestinal encephalopathy (MNGIE) disease (TYMP) [423]	*Lifetime implications*: Heterozygotes often have about 35% residual thymidine phosphorylase enzyme activity. Otherwise, they are expected to be asymptomatic.	<1 in 500	Limited
Molybdenum cofactor deficiency C (GPHN) [424]	*Lifetime implications*: There may be an increased risk of neurodevelopmental diagnoses, including schizophrenia, seizures, or autism spectrum disorder.	<1 in 500	Moderate
Mucolipidosis IV (MCOLN1) [425]	*Lifetime implications*: Lisch epithelial corneal dystrophy has been reported in heterozygotes of MCOLN1. Corneal bands of whorled, feathery, gray opacities characterize this. If the bands involve the central cornea, vision may be impacted.	1 in 300 Ashkenazi Jewish: 1 in 100	Limited
Neuronal ceroid lipofuscinosis (CLN6 and CTSD) [426, 427]	*Lifetime implications*: Obsessive‐compulsive disorder has been observed in CLN6 heterozygotes. More severe phenotypes, such as bipolar disorder, can follow a digenic inheritance pattern with the CLN6 gene. It has been proposed that haploinsufficiency of CTSD can result in Parkinson's disease, as a reduction in CTSD activity results in a decline of lysosomal functions and an increase in Lewy body pathology. Certain heterozygous variants in CTSD can cause early‐onset motor and visual disturbances, brain atrophy, and progressive psychomotor problems.	<1 in 500	Moderate
Niemann‐Pick disease (NPC1 and NPC2) [428, 429, 430]	*Lifetime implications*: NPC1 Heterozygotes may manifest deficits in cognition, particularly executive function, due to impaired cholinergic circuit. Parkinson's disease and progressive supranuclear palsy have also been observed in NPC1 heterozygotes. Corticobasal syndrome has been observed in NPC1 and NPC2 heterozygotes.	1 in 194	Moderate
Nijmegen breakage syndrome (NBN) [431, 432, 433, 434]	*Lifetime implications*: Pathogenic variants in NBN are associated with an increased risk of certain cancers, including breast, prostate, medulloblastoma, and melanoma, in carriers. *Clinical guidance*: Currently, the NCCN does not have guidelines for NBN heterozygotes. Therefore, more frequent or earlier screening is not recommended based solely on carrier status.	1 in 158	Limited
Nonsyndromic hearing loss 1A (GJB2) [435, 436, 437]	*Lifetime implications*: Two‐fold increase in hearing loss for those with the 35delG variant.	1 in 42 African population: 1 in 25 Ashkenazi Jewish: 1 in 21 European/Caucasian: 1 in 33 Latino: 1 in 100 Middle Eastern: 1 in 83 South Asian: 1 in 148	Moderate
Oculocutaneous albinism (TYR, OCA2, TYRP1, and SLC45A2) [438, 439]	*Lifetime implication*: Carriers may have lighter skin tone and eye color than relatives; increased risk for familial cutaneous melanoma.	TYR: 1 in 20 OCA2: 1 in 76 TYRP1: <1 in 500 African: 1 in 47 SLC45A2: 1 in 159 Japanese: 1 in 146	Limited
Odontoonychodermal dysplasia (WNT10A) [440, 441]	*Lifetime implications*: Non‐syndromic tooth agenesis is seen in some carriers with the absence of one or two permanent teeth.	<1 in 500	Moderate
Osteogenesis imperfecta type 15 (WNT1) [442, 443]	*Lifetime implications*: Carriers have been reported to have early onset osteoporosis.	<1 in 500	Moderate
Photosensitive trichothiodystrophy 1 (ERCC2) [556, 557, 558]	*Pregnancy concerns*: Carriers with an affected fetus have been noted to have poor outcomes, including fetal growth restriction, preterm birth, preeclampsia, and Hemolysis, Elevated Liver enzymes, and Low Platelets.	1 in 65	Limited
Primary hyperoxaluria type III (HOGA1) [444, 445]	*Lifetime implications*: Some carriers will have elevated urinary oxalate precursors. However, nephrolithiasis is not shown to be increased in carriers.	1 in 184	Limited
Primary microcephaly 1, recessive (MCPH1) [446, 447]	*Lifetime implications*: Carriers may have mild microcephaly, but are typically not impacted by other findings.	1 in 147	Limited
Progressive myoclonic epilepsy type 3 (KCTD7) [448]	*Lifetime implications*: There are reports that some carriers have an increased risk of developmental delays, seizures, autism and/or intellectual disabilities.	<1 in 500	Limited
Renal tubular acidosis (ATP6V0A4) [69, 70]	*Lifetime implications*: There can be a mild renal acidification defect without a decrease in blood pH, which can increase the risk for hypercalciuria and nephrolithiasis. Most carriers have mild symptoms but typically do not have distal renal tubular acidosis.	<1 in 500	Limited
Short stature, onychodysplasia, facial dysmorphism, and hypotrichosis syndrome (POC1A) [449, 450]	*Lifetime implications*: One case report of decreased POC1A levels in two heterozygotes. Other case reports demonstrated normal height.	<1 in 500	Limited
Sickle cell (HBB) [451, 452, 453, 454, 455, 456, 457, 458]	*Pregnancy considerations*: There is conflicting data on whether Sickle Cell Trait is associated with an increased rate of adverse outcomes, including preeclampsia, preterm birth, stillbirth, or venous thromboembolism. However, there is an established link between urinary tract infections and Sickle Cell Trait. *Lifetime implications*: While heterozygotes with hemoglobin A and S are typically asymptomatic since the HbS fraction is rarely above half, there are some circumstances where increased sickling can lead to vaso‐occlusive events, such as extreme physical exertion, dehydration, or altitude. There are rare reports of an increased risk for venous thromboembolism, exertional rhabdomyolysis, splenic infarcts at high altitude, and renal medullary carcinoma. *Clinical guidance*: General recommendations are to avoid dehydration and hyperthermia during strenuous exercise. There is conflicting data on the utility of performing urine cultures every trimester during pregnancy, but it is generally considered a low‐cost intervention.	1 in 158 African/African American: 1 in 10 East Asian: 1 in 50 Mediterranean: 1 in 3 South Asian: 1 in 25	Limited
SPG11‐related Neuromuscular Disorders (SPG11) [459]	*Lifetime implications*: In one case series, 4 of 7 heterozygotes had abnormal whitish ocular fundus. It is uncertain what the sequela of this finding are. *Clinical guidance*: Heterozygotes with symptoms should be evaluated by an experienced ophthalmologist.	1 in 159	Limited
Surfactant metabolism dysfunction, pulmonary 3 (ABCA3) [460]	*Lifetime implications*: A case series reported more frequent ABCA3 variants in children with unexplained chronic lung disease.	1 in 116	Limited
Three‐M Syndrome (CUL7) [461, 462]	*Lifetime implications*: While most carriers are asymptomatic, there are some reports of carriers with small stature, characteristic facies, or prominent talus.	<1 in 500	Limited
Thyroid dyshormonogenesis, TPO‐related (TPO) [373]	*Lifetime implications*: <10% risk of congenital hypothyroidism. *Clinical guidance*: Evaluate for congenital hypothyroidism	1 in 373	Limited
Vici Syndrome (EPG5) [463, 464, 465, 466]	*Lifetime implications*: There is one limited study that reported that heterozygotes had increased incidents of vitiligo, early‐onset cataracts, and some tumors. *Clinical Guidelines*: Symptomatic heterozygotes should be evaluated by an ophthalmologist. Currently, there are no dedicated recommendations for cancer screening; therefore, it should be guided by family history.	Ashkenazi Jewish: 1 in 224	Limited
Vitamin D‐dependent rickets, type 2A (VDR) [467]	*Lifetime implications*: There is a single case report of a monoallelic individual with vitamin D‐resistant rickets.	<1 in 500	Limited
Warsaw breakage syndrome (DDX11) [468, 469, 470]	*Lifetime implications*: DDX11 may act as a tumor suppressor. It is possible that carriers have an increased risk of malignancy. However, there are conflicting reports, and screening recommendations are unclear.	<1 in 500 Ashkenazi Jewish: 1 in 68	Limited
Wilson disease (ATP7B) [471, 472, 473]	*Lifetime implications*: Heterozygotes may exhibit laboratory findings that overlap with those of probands affected by the disease, including low serum ceruloplasmin concentrations, borderline urinary copper levels, and/or moderate hepatic copper elevation. Certain alleles have been associated with the disease in carriers.	1 in 87 Caucasian/European: 1 in 42 Ashkenazi Jewish: 1 in 70	Limited

^a^ Variants can be either autosomal dominant or autosomal recessive.

**TABLE 4:** X‐linked genes with moderate or limited evidence of causing risks for the carrier in the heterozygote state. Carrier frequency is described for the general population except where a specific ethnicity is described.

Condition (gene)	Pregnancy concerns/risks for female heterozygotes/clinical guidance	Carrier frequency (ethnicity) [13, 17]	Supportive evidence level [16]
Barth syndrome (TAZ) [474, 475, 476]	*Risks for female heterozygotes*: While cases of symptomatic females have been reported, most affected individuals have monosomy X mosaicism or are homozygous for the mutation. There are two case report of skewed X inactivation of the normal X chromosome in a symptomatic patient with hypotonia.	<1 in 50,000	Limited
Chondrodysplasia punctata type 1, X‐linked (ARSE) [477]	*Risk for female heterozygotes*: Some females may present with short stature.	1 in 250,000	Limited
Choroideremia (CHM) [478, 479, 480, 481]	*Risks for female heterozygotes*: After the age of 25, females often develop retinal pigment epithelial depigmentation or atrophy, which can present as night blindness or visual field loss.	1 in 250,000	Limited
Chronic granulomatous disease, X‐linked (CYBB) [482, 483]	*Risk for female heterozygotes*: Some carriers have an increased risk for inflammatory diseases.	1 in 149,254	Limited
Congenital adrenal hypoplasia, X‐linked (NR0B1) [484, 485]	*Risk for female heterozygotes*: Female carriers may have signs of hypogonadotropic hypogonadism, adrenal insufficiency, or delayed puberty.	1 in 6,250	Limited
Dent disease (CLCN5) [295, 296]	*Risks for female heterozygotes*: Most females present with low‐molecular‐weight proteinuria (70%), and half have hypercalciuria. Rarely, some female carriers will have a more severe manifestation that includes nephrolithiasis, and there are a few reports of end‐stage renal failure.	<1 in 500	Limited
Fanconi anemia group B (FANCB) [486]	*Risk for female heterozygotes*: Most females have skewed X‐inactivation of the affected X chromosome. However, there are a few cases of VACTERL with hydrocephalus reported in female carriers.	<1 in 50,000	Limited
Fragile XE syndrome (AFF2) [487]	*Risk for female heterozygotes*: Deletion at Xq27.3q28 may include FMR1 and AFF2, which can result in distinctive facial features and intellectual disability.	<1 in 50,000	Limited
Glycogen storage disease type IXa (PHKA2) [488, 489, 490, 491, 492]	*Risks for female heterozygotes*: Depending on skewed X inactivation, females may have mild hepatomegaly or, rarely, more severe symptoms such as short stature in childhood, biochemical abnormalities, and hypoglycemia. Polycystic ovarian syndrome has been reported.	<1 in 50,000	Limited
Hyper IgM syndrome, X‐linked (CD40LG) [493, 494]	*Risk for female heterozygotes*: Due to X‐inactivation, heterozygous females exhibit decreased levels of activated T cells. This can lead to an immune deficiency characterized by recurrent infections of the upper and lower respiratory tracts, low serum levels of IgG and IgA, and a deficiency in the in vivo antibody response.	1 in 50,000	Limited
Hypohidrotic ectodermal dysplasia (EDA) [495, 496, 497, 498]	*Pregnancy concerns*: Carriers of the X‐linked disorder should be counseled on proper nutrition during pregnancy. There is an ongoing phase 2 trial for affected male fetuses to receive intra‐amniotic injections of ER004. Mammary gland differences may impair breastfeeding. *Risks for female heterozygotes*: Females with an X‐linked allele have mosaic sweat glands that can be assessed using an iodine solution. It is common for female heterozygotes to have hypodontia (60‐80%).	1 in 14,167	Limited
Juvenile retinoschisis (RS1) [499, 500, 501]	*Risks for female heterozygotes*: Due to X‐inactivation, female carriers may experience bilateral inferotemporal retinoschisis, bilateral small central scotomas, and/or macular anomalies. Examination of the peripheral retina in female carriers may identify areas of schisis.	1 in 2,500	Moderate
L1 Syndrome (L1CAM) [502, 503]	*Risks for female heterozygotes*: Female heterozygotes may have adducted thumbs and/or mild intellectual disability. Due to X‐inactivation, some females may develop aqueduct stenosis or hydrocephalus.	1 in 15,000	Moderate
Lesch‐Nyhan Syndrome (HPRT1) [504, 505]	*Risks for female heterozygotes*: While females typically do not exhibit the motor or cognitive issues commonly seen in males, they may experience elevated uric acid levels, which can lead to gout at an older age.	1 in 150,000 [504, 505]	Limited
Lissencephaly, X‐linked (DCX) [506, 507, 508]	*Risks for female heterozygotes*: Heterozygous females are at risk of developing subcortical band heterotopia. This is characterized by ribbons of grey matter within the ventral white matter, located between the cortex and the ventricular surface. These patients may experience intellectual disability, language impairment, hypotonia, psychomotor delay, behavioral disturbances, or seizures.	1 in 42,500	Limited
Lujan‐Fryns syndrome (UPF3B) [509, 510]	*Risks for female heterozygotes*: At least one female heterozygote has been reported to have a marfanoid habitus and a high‐pitched nasal speech.	<1 in 50,000	Limited
Menkes disease (ATP7A) [511, 512, 513]	*Risks for female heterozygotes*: Due to X‐inactivation, variable clinical findings have been reported in heterozygote females. Clinical symptoms include pili torti, seizures, hypotonia, cerebrovascular tortuosity, intellectual disability, bladder diverticula, and connective tissue findings.	1 in 50,000	Moderate
Methylmalonic acidemia with homocystinuria, type cblX (HCFC1) [514]	*Risks for female heterozygotes*: Females can present with a milder version of the disease compared to their male counterparts. Findings can include dysmorphic features and intellectual and learning differences.	<1 in 50,000	Moderate
Myotubular myopathy (MTM1) [515, 516, 517, 518]	*Risks for female heterozygotes*: Due to X‐inactivation, female carriers may experience varying symptoms, ranging from severe, generalized weakness in children or infancy to mild weakness manifesting in adulthood. Asymmetric limb weakness, facial weakness, ptosis, and ophthalmoparesis have been observed. Affected adult females may also experience respiratory decline, which may require ventilatory support.	1 in 25,000	Moderate
Norrie disease (NDP) [519, 520, 521, 522, 523]	*Risk for female heterozygotes*: Depending on X inactivation, ocular manifestations can occur in heterozygous females, including retinal detachment, peripheral retinal avascularity, neovascularization, exudation, high hyperopia, vision loss, and mild sensorineural hearing loss.	<1 in 50,000	Moderate
Opitz GBBB syndrome, type I (MID1) [524, 525]	*Risks for female heterozygotes*: 50%–100% have isolated hypertelorism.	<1 in 50,000	Limited
Renpenning syndrome (PQBP1) [526, 527, 528]	*Risks for female heterozygotes*: Female carriers may have behavioral problems or dermatologic findings.	<1 in 250,000	Moderate
Wiskott‐Aldrich syndrome (WAS) [474, 529]	*Risk for female heterozygotes*: Depending on X‐inactivation, females may experience mild thrombocytopenia and neutropenia. In rare cases, female carriers can present with features of Wiskott‐Aldrich syndrome with severe thrombocytopenia and immune dysfunction.	1 in 250,000	Moderate
X‐linked Aarskog‐Scott syndrome (FGD1) [530, 531]	*Risks for female heterozygotes*: Due to X inactivation, many female carriers exhibit mild features, such as brachydactyly, hypertelorism, short stature, and a widow's peak hairline.	<1 in 50,000	Moderate
X‐linked agammaglobulinemia (BTK) [532, 533, 534]	*Risks for female heterozygotes*: There are case reports of skewed X inactivation, characterized by a lack of peripheral B cells or variations in respiratory disease expressivity.	<1 in 50,000	Moderate
X‐linked intellectual disability syndrome 34 (NONO) [535, 536]	*Risks for female heterozygotes*: There have been reported cases of intellectual differences in female carriers; however, most females appear to be unaffected.	<1 in 50,000	Moderate
X‐linked intellectual disability, AP1S2‐related (AP1S2) [537, 538, 539]	*Risk for female heterozygotes*: There is one report of a female with intellectual disability. The remaining case series describe unaffected female carriers.	<1 in 50,000	Limited
X‐linked intellectual disability, BRWD3‐related (BRWD3) [540, 541]	*Risks for female heterozygotes*: Females with skewed X inactivation can be affected. They typically present with epilepsy, and sometimes also have macrocephaly and learning differences.	<1 in 50,000	Moderate
X‐linked intellectual disability, CUL4B‐related (CUL4B) [542, 543, 544]	*Risk for female heterozygotes*: Some females are reported to have mildly dysmorphic features like ear lobe abnormalities in addition to language and learning differences, attention deficit disorder, tremors, tics, or alopecia.	<1 in 50,000	Limited
X‐linked intellectual disability, IL1RAPL1‐related (IL1RAPL1) [545, 546]	*Risks for female heterozygotes*: There are reports of possible learning differences.	<1 in 50,000	Moderate
X‐linked intellectual disability, PAK3‐ related (PAK3) [547, 548]	*Risks for female heterozygotes*: A case series has shown that females may sometimes present with cognitive differences. In other reports, females have mild ichthyosis on extensor surfaces.	<1 in 50,000	Moderate
X‐linked intellectual disability, Siderius type (PHF8) [549]	*Risks for female heterozygotes*: Some females are reported to have intellectual differences and epilepsy.	<1 in 50,000	Limited
X‐linked intellectual disability, THOC2‐related (THOC2) [550, 551]	*Risks for female heterozygotes*: There are reports of females with intellectual differences.	<1 in 50,000	Limited
X‐linked intellectual disability, ZDHHC9‐related (ZDHHC9) [552]	*Risks for female heterozygotes*: There are reports of females with intellectual differences.	1 in 4,999,901	Moderate
X‐linked intellectual disability, ZNF711‐related (ZNF711) [553]	*Risks for female heterozygotes*: There are reports of females with intellectual differences.	<1 in 50,000	Limited
X‐linked ocular albinism, GPR143‐related (GPR143) [554, 555]	*Risks for female heterozygotes*: Iris transillumination defects are seen as “mud‐splattered” retinal appearances. About 50%–100% of female heterozygotes will have retinal differences.	1 in 25,000	Limited
X‐linked retinitis pigmentosa, RP2‐related (RP2) [142, 346, 347, 348]	*Risks for female heterozygotes*: Depending on X‐inactivation, females can be unaffected or express mild to severe retinal degeneration.	1 in 4,000	Moderate

### 2.4 Conditions with clinical implications in the carrier state

We want to highlight some of the most important autosomal recessive conditions with clinical implications in the carrier state that can impact patients during pregnancy.

#### 2.4.1 Neurologic conditions

There are several conditions associated with Parkinson's disease and Parkinson's‐like presentation. The most common is Gaucher disease (GBA, definite evidence), where nearly 10% of carriers develop Parkinson's disease by age 80 [107, 108, 109, 110]. There is moderate evidence that carriers of Neuronal ceroid lipofuscinosis (CLN6 and CTSD) [426, 427] and Niemann‐Pick diseases (NPC1 and NPC2) [428, 429, 430] may also have an increased risk of Parkinson's disease.

#### 2.4.2 Cardiovascular disorders

Patients who are carriers of dystrophinopathies, including DMD (definite evidence), are at increased risk for dilated cardiomyopathy and should undergo an echocardiogram every 5 years [8, 9, 10, 11]. Fabry disease (GLA, definite evidence) is also linked to cardiovascular disease, and carrier patients should be evaluated for renal and cardiac sequelae [274, 275, 276].

#### 2.4.3 Respiratory conditions

Carriers of alpha‐1 antitrypsin deficiency (SERPINA1, definite evidence) should be counseled on tobacco cessation due to their increased risk of lung disease and emphysema [23, 24]. Cystic fibrosis (CFTR, definite evidence) carriers may be at an increased risk for pulmonary and pancreatic complications [57, 58, 59, 60, 61, 62, 63].

#### 2.4.4 Renal conditions

Heterozygous carriers of familial Mediterranean fever (MEFV, definite evidence) who are symptomatic should be treated with colchicine [102, 103, 104].

#### 2.4.5 Hematologic disorders

Hematologic disorders are common in the carrier state, and many patients benefit from hematology referrals. Factor XI (F11, definite evidence) [89], Glanzmann Thrombasthenia (ITGA2B, ITGB3, definite evidence) [113, 114, 115], Hemophilia A (F8, definite evidence) [283, 284], and Hemophilia B (F9, definite evidence) [284, 285] have an increased risk for bleeding at the time of delivery and may require tranexamic acid or factor replacement, depending on the disorder.

#### 2.4.6 Cancer predisposition

At the time of publication, only three genes implicated in cancer predisposition have specific National Comprehensive Cancer Network (NCCN) guidelines, despite several other genes that may pose a risk for heterozygotes. Ataxia‐telangiectasia (ATM, definite evidence) c7271T > G variants have a risk of breast cancer similar to that of Breast Cancer Type 1 or 2 susceptiability genes (BRCA1 and BRCA2) pathogenic mutations and guidelines recommend regular breast cancer screening starting at age 25 [27, 28, 29, 30]. Carriers of Fanconi Anemia Group J (BRIP1, definite evidence) should have the option to undergo a prophylactic salpingo‐oophorectomy at age 45–50 [27]. Fumarase deficiency (FH, definite evidence) carriers should start yearly abdominal imaging for renal cell malignancy screening at age 8 to 10 [105, 106].

Most of the mutations that have cancer predisposition do not have specific guidelines at this time. For individuals who are carriers of Bloom syndrome (BLM, limited evidence) [361, 362], Dyskeratosis congenita type 4 (TERT, definite evidence) [80, 81], Dyskeratosis congenita type 5 (RTEL1, definite evidence) [82, 83, 84], Fanconi Anemia, Group A (FANCA, moderate evidence) [390], Nijmegen breakage syndrome (NBN, limited evidence) [431, 432, 433, 434], and Vici syndrome (EPG5, limited evidence) [463, 464, 465, 466], cancer screening should be guided by family history. These patients may benefit from referral to a provider with expertise in cancer genetics.

### 2.5 Conditions with implications during pregnancy

We identified nineteen conditions that raise specific concerns during pregnancy. Obstetric clinicians should pay special attention to patients found to be carriers of these conditions during pregnancy.

*Alport syndrome COL4A5‐related (COL4A3, COL4A4, COL4A5*, *definite evidence)* carriers often have renal sequelae, including microhematuria and proteinuria. These patients should undergo a baseline urine protein assessment and be closely monitored for the development of preeclampsia. A referral to nephrology can be considered in the setting of nephrotic range proteinuria [258, 259, 260].

*Dubin Johnson syndrome (ABCC2, moderate evidence)* carriers have an increased risk for intrahepatic cholestasis during pregnancy. They should be monitored for the disease during pregnancy and lifelong monitoring for the development of hepatic disease [186, 385].

*Carnitine palmitoyltransferase 1A deficiency (CPT1A, strong evidence)* is a disorder of long‐chain fatty acid oxidation. If the fetus is homozygous for this condition, then there is an increased maternal risk of acute fatty liver. In the absence of an affected fetus, there are no identified reports of maternal disease in heterozygotes [39, 40, 41].

*Ehlers‐Danlos‐like syndrome due to tenascin‐X deficiency (TNXB, moderate evidence)* heterozygous carriers can have a decrease in TNX serum levels, and caution should be taken with emergency intubations due to the risk of esophageal rupture [387, 388, 389].

*Fabry disease (GLA*, *definite evidence)* is linked to cardiovascular disease, and carrier patients should be evaluated for renal and cardiac sequelae. These patients also have an increased risk of hypertension and postpartum depression [274, 275, 276].

*Factor V Leiden (F5*, *definite evidence)* carriers have an increased risk of venous thromboembolism. Depending on risk factors, family history, and personal history, anticoagulation may be recommended during pregnancy or postpartum [87, 195].

*Factor XI (F11, definite evidence)* [89], *Glanzmann Thrombasthenia (ITGA2B, ITGB3, definite evidence)* [113, 114, 115], *Hemophilia A (F8, definite evidence)* [283, 284], and *Hemophilia B (F9, definite evidence)* [284, 285] have an increased risk for bleeding at the time of delivery and often require tranexamic acid or factor replacement, depending on their factor levels and personal/family history. These patients should have their factor levels assessed during the third trimester and may be referred to a hematologist.

*Familial hypercholesterolemia (LDLR*, *definite evidence)* carriers who are managed on statins should be switched to bile acid‐binding resins during pregnancy because of the safer pregnancy profile [91, 92, 93, 94, 95].

*Glucose‐6‐phosphate dehydrogenase deficiency (G6PD*, *definite evidence)* carriers should avoid high doses of primaquine and fava beans. If there is an affected male fetus, pharmacologic or chemical exposures, such as consumption of medications and other exposures that are unsafe for people with G6PD, should be avoided during pregnancy and lactation [279, 280, 281, 282].

*Ornithine transcarbamylase deficiency (OTC*, *definite evidence)* is essential for obstetricians to be aware of since carriers can have encephalopathic episodes during the peripartum period. Ammonia levels should be closely monitored during delivery and treated as needed. Life‐threatening catabolic episodes have been reported in carriers, and these patients require multidisciplinary management [307, 308, 309].

*Photosensitive trichothiodystrophy 1 (ERCC2, limited evidence)* carriers with an affected fetus have been noted to have poor outcomes, including fetal growth restriction, preterm birth, preeclampsia, and hemolysis, elevated liver enzymes, and low platelets [556, 557, 558].

*Progressive familial intrahepatic cholestasis (ABCB11, ABCB4*, *definite evidence)* carriers have an increased risk for intrahepatic cholestasis during pregnancy. They should be monitored for symptoms during pregnancy and lifelong for the development of hepatic disease [186, 187].

*Sickle cell trait (HBB, moderate evidence)* carriers have an increased risk of urinary tract infections. There is conflicting data on the utility of performing urine cultures every trimester during pregnancy, but it is generally considered a low‐cost intervention [451, 452, 453, 454, 455, 456, 457, 458].

*Trifunctional protein deficiency (HADHA, HADHB*, *definite evidence)* can have consequences for pregnant persons with an affected fetus. If the fetus is affected, there is a 15%–62% risk of maternal HELLP syndrome or acute fatty liver of pregnancy. Even in the absence of an affected fetus, heterozygous pregnant females are at an increased risk for HELLP or acute fatty liver of pregnancy [225, 226, 227, 228, 229, 230, 231, 232].

*X‐linked dyskeratosis congenita (DKC1*, *definite evidence)* females are at risk for pancytopenia depending on skewed X inactivation and should be monitored for bone marrow failure [330, 331].

*17‐Beta hydroxysteroid dehydrogenase (HSD17B3, limited evidence)* may increase preeclampsia risk, though data are limited [353].

### 2.6 Pretest counseling considerations

Patients undergoing carrier screening should be informed that the purpose of carrier testing is to predict recessive conditions in future offspring. Some of the conditions are caused by genes on the X chromosome (X‐linked recessive conditions). If a female is a carrier, offspring will have a 50% risk of inheriting the affected X chromosome. For autosomal recessive conditions, if both partners are carriers for the same condition, then there is a 25% risk for an affected offspring. In some cases, being a carrier has medical implications, and this may be important information for their medical management. It is also important to review interventions available if a fetal risk is identified. Interventions can include preimplantation genetic diagnosis or prenatal testing. For at‐risk couples who are already pregnant or who choose not to pursue risk‐reducing interventions, knowing that a newborn is at risk would prompt neonatal testing with the opportunity to pursue treatment or interventions. It has been estimated that about 8.3% of couples who undergo expanded carrier testing are found to be carrier couples that have a reproductive risk [559].

Patients should also be informed of the limitations of expanded carrier screening, including that not all genetic conditions can be predicted in advance [3]. Most labs report only pathogenic and likely pathogenic variants. Variants of unclear significance are not reported but may be pathogenic. Thus, if the patient or partner screens negative for variants via a pan‐ethnic panel, this reduces but does not eliminate the possibility of fetal disease risk. In addition, some genetic diseases, like Duchenne muscular dystrophy, commonly occur de novo and are not always inherited from a carrier parent [269]. Therefore, a residual risk of being a carrier for a rare genetic condition always remains despite a negative screening result.

Patients should be counseled about the option of carrier screening for autosomal recessive conditions. This conversation should include the fact that all healthy individuals can be carriers of recessive and/or X‐linked conditions. Predicting a fetal risk prior to conception would allow a couple to minimize the chance of having an affected child with the aid of preimplantation genetic testing and/or prenatal testing. For some conditions, treatment in utero or after birth is available, and knowing in advance which child is at risk provides an opportunity for neonatal management and treatment, especially for conditions that are not on the newborn screen. Patients should also be advised that in about 9% of cases, there may be direct implications for the proband from carrier status alone [6]. The limitation of carrier screening is that not all genetic conditions can be predicted. The technology does not predict 100% of carriers; thus, testing the parents does not guarantee an unaffected fetus.

### 2.7 Posttest counseling considerations

The ordering physician should review the carrier screen report carefully, noting any known medical implications associated solely with the carrier state. At times, the implications for the carrier may be specific to the identified variant. Carefully reading the report, including the section on the identified variant, will provide guidance on how to counsel the patient. A referral for genetic counseling should be considered for patients at risk of clinical implications in the carrier state. In addition, any female who is an X‐linked carrier or a couple at risk for a child with an autosomal recessive condition should be referred for genetic counseling to discuss the natural history of the condition and reproductive interventions available. Once a carrier is identified, it is essential to discuss how this result may affect family members and the importance of sharing their results/report. Partners should also be offered expanded carrier screening, especially for any variants identified on the pregnant person's report. Fetal testing by chorionic villus sampling or amniocentesis should be offered to appropriate patients.

Since manifestations in carriers for recessive or X‐linked conditions are strongly variant‐dependent, it is our opinion that the reporting laboratory should be required to comment on specific variants associated with carrier manifestations and the likelihood of such manifestations.

## 3 CONCLUSIONS

There are clinical implications for carriers of some single‐gene disorders included in prenatal carrier screens. Many conditions can cause complications during pregnancy or later in life. During pretest counseling, providers ordering carrier screening should discuss the possibility of uncovering carriers who are manifesting the condition. Nevertheless, the limitations of this review should be stressed. In some rare variants, there is a scarcity of published cases. If a provider identifies a patient who is a carrier of a condition with possible manifestations in the heterozygote state, they should review the exact variant the patient has to see if it has implications in the carrier state. Often, this information can be found in the laboratory report.

As these panels continue to include a larger number of genes, the ordering provider must review the results in the context of the gene and the condition reported by the lab they utilize. Molecular carrier screening panels may disclose valuable health information for the proband and their family, beyond reproductive risk assessment, and can have direct implications on their obstetrical care and management.

## CONFLICT OF INTEREST STATEMENT

The authors declare no conflicts of interest.

## FUNDING INFORMATION

The authors received no specific funding for this work.

## Data Availability

Data sharing is not applicable to this article as no datasets were generated or analyzed during the current study.
